# Donald Trump’s grammar of persuasion in his speech

**DOI:** 10.1016/j.heliyon.2019.e03082

**Published:** 2019-12-26

**Authors:** Achmad Fanani, Slamet Setiawan, Oikurema Purwati, Maisarah Maisarah, Uswatun Qoyyimah

**Affiliations:** a Universitas Negeri Surabaya, Universitas Pesantren Tinggi Darul Ulum Jombang, Indonesia; b Universitas Negeri Surabaya, Indonesia; c Universitas Pesantren Tinggi Darul Ulum Jombang, Indonesia

**Keywords:** Linguistics, *Ethos*, *Pathos*, *Logos*, SFL mood analysis, Speech

## Abstract

This article presents an analysis of the nature of propositions made in President Trump's persuasive, yet controversial speech on Jerusalem from the perspective of mood analysis. The interpersonal relationships between the speaker and the audience concerning the building of *ethos, pathos,* and *logos* are revealed. It applies a discourse analysis with a qualitative approach to see how the President grammatically composed his *ethos, pathos,* and *logos* clauses. The results show that in the speech: 1) the *ethos* clause was built by employing the declarative mood functioning as a statement to show his credibility; 2) the *pathos* clauses were composed by implementing two moods: mostly declaratives which mainly functioned as statements, and few imperative moods to arouse both positive and negative feelings of the audience; 3) and the *logos* clauses were composed by using the declarative moods functioning as statements to give bases for his argumentation. The high use of declarative moods indicated that he positioned himself as an information bearer, to shorten the gap between him and his audience. Grammatically, the controversial side of the speech was mostly featured by several clauses containing negative elements such as blaming and negative polarity, especially when talking about previous US presidents and governments.

## 1 Introduction

This paper is an investigation into the nature of propositions made in President Trump's speech on Jerusalem delivered on December 6, 2017 (Trump, 2017), from the perspective of Systemic Functional Linguistics (SFL). The speech was a persuasive yet controversial one. It was persuasive since he used it to persuade the audience to agree with his decision. More specifically, this speech can be categorized as an analytical exposition whose purpose is to persuade that something is the case (Coffin, 2004). The speech was also controversial because it had received a positive response from the President's supporters but got adverse reactions from his rivals which strengthened tensions across the Middle East (Sofos and Felci, 2017). Because of its persuasive and controversial values, the speech is worth analyzing.

The primary purpose of a persuasive speech is to get the audience convinced and persuaded about the subject matter of the speech. The grammatical analysis on a persuasive speech is worthwhile to do because one's techniques of persuading the audience can be seen from the grammatical choices the person uses, as grammar is vital in persuasion (Power, 1998). The grammatical viewpoint was chosen to analyze the rhetorical device of *ethos, pathos,* and *logos* because most of the previous studies concerned with the discussion of the power words, pragmatics, social, even psychological aspects of the devices (e.g., Braet, 1992; Higgins and Walker, 2012).

This current research carries out a lexico-grammatical analysis on the main clauses in President Trump's persuasive yet controversial speech with the aim of identifying how interpersonal relationships are created between the speaker and the audience and how the systems of mood are used to build the *ethos*, *pathos*, and *logos* (Aristotle's elements of persuasion). Wardhaugh (2006, p. 104) explained that “a change of topic requires a change in the language used” which implies that different purposes of speech will require a different linguistic strategy to apply. For example, to have someone to take medicine will likely need different linguistic strategies than to have the same person to have ice cream. It would be interesting, therefore, to analyze how the President built his *ethos*, *pathos*, and *logos* clauses in his political speech.

The three elements (*ethos*, *pathos*, and *logos*) are essential for persuading the audience on a given topic in a persuasive speech. One of the factors contributing to the successful building of the elements lies in the mood system being used. It means that the right mood system to apply will affect the persuasiveness of the message the sender intends to deliver. The result of the analysis is to give a comprehensive view of how *ethos*, *pathos*, and *logos* were built structurally in a persuasive, yet provocative speech.

## 2 Literature review

### 2.1 Definition of *ethos*, *pathos*, and *logos*

The words *ethos, pathos,* and logos come from the Greek language. *Ethos* (the ethical appeal) is used by a speaker to convince the audience about the speaker's credibility or character (Aristotle, as cited in Kennedy, 1991; Braet, 1992). *Ethos* means “character”. By the *ethos*, a speaker demonstrates that he is a trustworthy source of information and therefore should be listened. To build an *ethos*, a speaker can choose appropriate language for the audience. It can be done by making him/her seem or sound rational or fair, showing his/her capability or expertise, and using correct grammar and syntax (Higgins and Walker, 2012). The following sentence is an example of *ethos*, “My three decades of experience in public service, my tireless commitment to the people of this community, and my willingness to reach across the aisle and cooperate with the opposition, make me the ideal candidate for your mayor” (Bernanke, 2010).

*Pathos* means “suffering” and “experience”, and is also known as the emotional appeal. It is generally used by a speaker to persuade an audience by appealing to the emotions or sentiments of the audience (Aristotle, as cited in Kennedy, 1991; Braet, 1992). *Pathos* is used to raise empathy from an audience, making the audience feel what the speaker would like them to feel. In short, *pathos* deals with the appeal to the audience's emotions which, according to Plutchik (1997), consist of 8 types of emotions: fear, anger, sadness, joy, disgust, surprise, trust, and anticipation. Usually, *pathos* is created by a speaker by arousing a pity as well as irritation from an audience; perhaps to speed action. A speaker can develop *pathos* by using meaningful language, emotive tone, emotion arousing instances, stories of sensitive events, and indirect meanings. The following is an example of *pathos*: “Peace of mind is the most important one. Our innovative security systems will secure the comfort of your family so that you can sleep peacefully in the night.”

*Logos* is the appeal to logic (Aristotle, as cited in Kennedy, 1991; Braet, 1992) and is used for persuading an audience by using logic or reasons. *Logos* can be formed by quoting facts and statistics, historical and literal analogies, and citing convinced authorities on a subject. *Logos* can be developed by using advanced, theoretical or abstract language, citing facts (very important), using historical and literal analogies, and constructing logical arguments. The following is an example of *logos*: “The data is completely perfect: this venture has reliably turned a profit year-over-year, even despite market drops in other regions.”

Previous studies on *ethos, pathos,* and *logos* suggest that the rhetorical elements played a critical role in the success of public speaking. Fengjie et al. (2016), for example, revealed several factors that supported the success of Obama's speech, one of which was various rhetorical devices in his speeches. They identified seven rhetorical elements applied in Obama's speech that reflect *ethos*, *pathos*, and *logos* propositions: alliteration, simile, metaphor, metonymy, synecdoche, antithesis, and parallelism. Ko (2015) found out that *ethos*, *pathos*, and *logos* were widely used in Taiwanese President Ma's political discourse on the cross-Straits Economic Cooperation Framework Agreement (ECFA). One of the interesting results of the study showed that Ma's *pathos* is abundantly filled with the negative elements of fear and anger, and positive elements of hope and security, especially in the question-and-answer session.

However, like the works of Fengjie et al. (2016) and Ko (2015) most of the linguistic research focus merely on the semantic level than on the grammatical level, although grammar is also a determinant factor in persuasion (Power, 1998). Because of its importance, analyzing the grammar of a public speech would be worthwhile. One of the prominent tools for analyzing grammar is Hallidayan SFL (Systemic Functional Linguistics) because it deals with interpersonal meaning or mood system, that is, how language is used concerning the relationship with other people.

### 2.2 Mood system in SFL

The concept of Systemic Functional Linguistics was first introduced by Halliday in the 1960s in the United Kingdom, and later in Australia. It is made as a grammar model that sees language as a set of semantic choices (Bloor and Bloor, 2004), which means people use language choices to produce meanings. The choice of different words and other syntactic or grammatical features will also have different meanings.

One of the metafunctions in SFL is the interpersonal metafunction. It is related to the social world, especially the relationship between the speaker and the listener (Halliday, 2014). Interpersonal metafunction regards clauses as exchanges. It can be described by explaining the semantics of interaction and the metalanguage that correlate with language as interaction (exchange) and modality. In this regard, this current study suggests that speakers/writers must be able to use language in such a way as to position themselves before their audience/readers. Hence, this analysis focuses on the interpersonal meaning implied in a speech to see how the speaker uses his speech to persuade the audience because persuasion is closely related to the relationship between the speaker/writer and the audience/readers.

The mood system has two basic terms: imperative and indicative (Halliday, 2014; Eggins, 2004). The indicative clause is related to the exchange of information (proposition negotiation), while the imperative clause relates to the performance of an action to provide services or to exchange goods (proposal negotiation).

The indicative mood is divided into two: declarative and interrogative. Although both declarative and interrogative contain elements of tense, person, and number, they have syntactically and semantically different forms. Declaratives have the typical speech-function realization as statements (facts, opinion, etc.) that serve to provide information; while interrogatives are the mood of the question that serves to request information (Halliday, 2014; Eggins, 2004).

The imperative mood is the mood of the verb and “the principal mood of will and desire” (Lyons, 1977). This mood is characterized by a verbal group in the form of a basic form of a verb. Imperatives have typical speech-function realization as orders, requests, and directives (Eggins, 2004; Emilia, 2014). The imperative mood does not occur in subordinate clauses or subordinate questions because basically, this kind of mood is performative (Palmer, 2001).

In the semantic of interactions, clauses are used to analyze how the language is used to connect with others, negotiate relationships, and to express opinions and attitudes. According to Halliday (2014), the relationship between speakers is made whenever the language is used to connect with other people. Halliday further explained that there are two basic types of speech roles: giving and demanding. Giving means inviting to accept, for example, ‘Do you want to have this book?’ On the other hand, demanding means inviting to give, for example, ‘Can I have the book?’ In the case of commodity exchange, Information and Goods & services are two types of commodities exchanged.

Each type of mood includes different constituent structures. In this case, the complete English clause has several functional elements, namely Subject, Finite, Predator, Complement, and Adjunct. The type of mood of the clause is determined by the subject and finite position in a clause, while the clause residue is filled by a combination of Predicator, Complement, and Adjunct.

Systemic Functional Linguistics (SFL) is commonly used as the approach to analyze the functional meaning of a language. Many researchers had applied SFL from different dimensions and perspectives. Kamalu and Tamunobelema (2013) used SFL to analyze religious identities and ideologies construed in a literary text. They found out that SFL Mood analysis was useful to understand the structural based interpersonal relationships of the participants in the literary text. Ayoola (2013) analyzed some political adverts of two parties in Nigeria concerning the interpersonal metafunction (mood system). One of his highlighted findings is that the interpersonal meaning of a structure does not always correspond with its lexicogrammar analysis. The writers used different mood types to interact, negotiate, and establish their relationship with the readers. The mood system was also applied to change the readers’ behavior. Ayoola concluded that contextual factors (e.g., the need to reflect the economic and socio-political context/situation of the country) profoundly influenced the mood types used in the adverts as well as their interpersonal meanings.

## 3 Method

This current research was a discourse analysis applying a qualitative approach to see how President Trump grammatically composed his *ethos*, *pathos*, and *logos*. The data source (the President's speech about Jerusalem) was taken from www.whitehouse.gov. In his speech, 74 sentences consisted of 71 major clauses and eight minor clauses. In this research, each simple sentence or complex sentence was counted as one clause. One compound sentence consisting of two major clauses was calculated as two clauses, depending on the number of main clauses that construct the sentence.

The clauses were then classified into each element of persuasion (*ethos*, *pathos*, *logos*). As the data, one clause belonged to *ethos* element; 50 clauses dealt with *pathos*, and 20 clauses referred to *logos*. The analysis was conducted by exploring the types of mood and speech functions as well as the mood elements (subject, finite, predicator, complement, and adjunct) that supported the elements of persuasion (*ethos*, *pathos*, *logos*).

## 4 Results and discussion

### 4.1 Results

The mood type and speech-function realization of his *ethos* clause can be described in Table 1.

**Table 1:** Mood type and speech-function realization in President Trump's *ethos* clause.

Mood Type	Speech function realization	Technique	Example
Declarative	Statement of fact	Presenting a personal experience (*I did y positively*)	(a) *“When I came into office, I promised to look at the world's challenges with open eyes and very fresh thinking.” (Clause 2)*

President Trump only used one clause (a) to build credibility (*ethos*) and was delivered in the declarative mood. The declarative mood functioned as a statement of fact and was composed by presenting a personal experience. It indicates that his main concern was to make a statement by providing convincing information about his credibility in addressing the topic of the speech. In this instance, he presented himself as a person with clear and unbiased thinking. The aim was to make the readers believe that the decision he would make through his speech would be true and reasonable.

The subject of the clause was a personal specific subject in the form of the personal pronoun “I”. He used “I” to tell the audience that it is “he”, not the other else, who did something which implies his credibility. Through the ethos clause, he would like to say that it is he who made the promise to look at the world's challenges with open eyes and very fresh thinking.

In communicating his self-credibility, the finite element of the clause used positive polarity. Such polarity gives a positive validity of the proposition and helps him create positive nuance to the audience about him. The positive polarity would make the audience directly understand that 'yes, he did it' (that he promised something).

In building the ethos clause, President Trump made use of an adjunct as the theme of the clause, putting them in the front part of the clause. He began the clause with the circumstantial adjunct “When I came into office” that directly orients the audience to the very first time he became the President of the US. The other circumstantial adjunct reflects the core of *ethos* that he built. The phrase “with open eyes and very fresh thinking” convinces the audience that he does everything objectively with an unbiased decision, including his decision to move the US embassy to Jerusalem.

In appealing to the audience's emotions (*pathos*), President Trump used more clauses which were dominated by declarative moods and, in a few occasion, imperative ones. The speech function realization of the declarative moods varies across clauses (as statements of opinion, as statements of assertion, and as indirect requests). However, the imperative mood was used in line with its typical function that is a direct request. The description of mood type and speech-function realization of his *pathos* clause can be seen in Table 2.

**Table 2:** Mood type and speech-function realization in President Trump's *pathos* clauses.

Mood type	Speech Function Realization	Technique	Example
Declarative	Statement of opinion	Presenting an evaluative opinion	(b) *We cannot solve our problems by making the same failed assumptions and repeating the same failed strategies of the past. (Clause 3)*
	Statement of assertion	Affirming the decision Asserting a commitment Asserting the expectation Predicting an impact	(c) *It's something that has to be done. (Clause 39)* (d) *The United States would support a two-state solution if agreed to by both sides. (Clause 50)* (e) *“Above all, our greatest hope is for peace, the universal yearning in every human soul.” (Clause 52)* (f) *“There will, of course, be disagreement and dissent regarding this announcement.” (Clause 54)*
	Statement of inclination	Asserting an intention of doing something	(g) *“I intend to do everything in my power to help forge such an agreement.” (Clause 50)*
	Indirect request	Asserting a request to do something	(h) *“And finally, I ask the leaders of the region — political and religious; Israeli and Palestinian; Jewish and Christian and Muslim — to join us in the noble quest for lasting peace.” (Clause 70)*
Imperative	Direct request	Request with let's	(i) *“Let us rethink old assumptions.” (Clause 68)*

In his *pathos* clauses, President Trump employed two kinds of mood: declarative and imperative moods. In these clauses, the declarative moods were dominantly used to function as statements. However, some of them were applied differently to make an indirect request to the audience as in (h). Another mood, imperative, was used to make a direct request to the audience as in (i).

The declarative moods indicating *pathos* clauses functioned differently. At least four speech-function realizations of his declarative moods were identified. *Firstly*, it functioned as statements of opinion which commonly pointed to his belief or judgment about something or someone. The technique employed was presenting an evaluative opinion as in (b). In this instance, he used a negative declarative mood to evaluate the previous assumptions and strategies made by the previous presidents, which, according to him, were totally failed to solve the problem.

Besides, the phrases ‘failed assumptions’ and ‘failed strategies’ in (b) clearly indicated his negative evaluative opinion (blaming) on the previous US governments' policies. Similar clauses criticizing or blaming previous US presidents' policy on Israel-Palestinian conflict also existed. It seems that the criticisms were utilized as the entrance door to pose the 'new' method he would like to deliver in solving the Israel-Palestinian conflict.

*Secondly*, the declarative mood in President Trump's speech functioned as a statement of an assertion. Through the clauses, he would like to show the audience that his decision is right and would result in a good impact. There were four techniques used: (1) Affirming the decision, (2) Asserting a commitment, (3) Asserting the expectation, and (4) Predicting an impact.

The first technique, affirming the decision, can be seen in (c). In this instance, President Trump assured the audience that relocating the embassy to Jerusalem was the right decision. He touched the audience's emotion by using the modality 'has to' which indicates something imperative to do. In his speech, the main objectives of affirmation were making assertions that ‘It is the right decision’ and ‘It is the best time to do it’.

The second technique is asserting a commitment to solving the problem as exemplified in (d). In this instance, he communicated to the audience that his government committed to supporting the two-state solution as long as the two parties agreed. This clause was clearly used to arouse the audience's positive attitude toward the US's commitment to solving the Israel-Palestinian conflict.

The third technique is asserting an expectation as in (e). Through this technique, an emotion of anticipation (looking forward positively to something which is going to happen) was built. In this instance, President Trump presented his hope regarding the relocation of the US embassy to Jerusalem. In his opinion, such kind of decision would result in peace.

The fourth technique is predicting an impact as in (f). This technique is utilized to answer the possible audience's question of ‘what would be the effect of the decision?’ In this instance, he predicted that the decision would soon invite a dissenting opinion from some parties. This clause would satisfy the audience's question of whether President Trump had anticipated the impact or not, specifically the negative one.

*Thirdly*, some of the declarative moods function as a statement of inclination as exemplified in (g). In this instance, President Trump's assertion “I intend to do everything in my power” was used to convince the audience that he would do his best in solving the problem. This clause is a strong personal inclination that may arouse the audience's positive feeling (i.e., trust) that he would be able to achieve his objective because he would use any resources to do it.

*Fourthly*, lexicogrammatically, President Trump's declarative mood, however, was not always aimed at giving information (statements) to the audience but also requesting someone to do something implicitly. In some parts of his speech, he sought for audience's agreement to do the great thing (i.e. creating peace in the Middle East). The clause (h) indicates his request to the leaders of the regions to join him in the noble quest for lasting peace. In other words, he asked the audience to agree with him that expelling the extremists from their midst was the best way to create peace in the Middle East.

Besides the declarative moods, three clauses were delivered in imperative moods to make a direct request to the audience to do something as can be seen in (i). The technique applied was a request with ‘let us’. The choice of using the inclusive ‘us’ was intended to make the audience feel involved and become closer to the speaker.

In the case of the elements of moods employed for touching the audience's emotion, three pronouns were frequently used as the subject of the clause (we, I, and impersonal ‘it’). President Trump used the inclusive ‘we’ 2 times and exclusive ‘we’ 4 times. The inclusive ‘we’ was used to involve the audience in the same problem as in “We cannot solve our problems …” (clause 3). In this instance, “we” refer to the audience and President Trump's government indicating that the problem faced was his as well as the audience's problem. On the other hand, the exclusive ‘we’ was used to refer to the US government only as in “We are not …borders” (clause 42). Here, the exclusive ‘we’ was utilized to assert to the audience about his government's position on the problem.

Another interesting use of the pronoun in building *pathos* clauses is the impersonal ‘it’ as the subject of the clause, which was employed three times consecutively. It was used to emphasize the importance of the present time to do something. For example, in “it is time …midst” (clause 62), President Trump told the audience that “it is the best moment” for everyone who wants peace to expel the extremists.

President Trump utilized mostly positive polarity in building *pathos*, but some of the clauses used negative ones. He commonly used positive polarity, especially when talking about the purpose of his decision, Israel, and the Middle East. Meanwhile, he also frequently used negative polarity when talking about previous US presidents. He not only used ‘not’ but also negative-sensed words to indicate negative polarity as in “While previous presidents …failed to deliver” (clause 15). The word ‘failed' clearly indicates a negative sense (as opposed to ‘success’) because, in this instance, it refers to the unsuccessfulness of the previous presidents in solving the problem.

The tense in his *pathos* clauses varied from past, present, and future times. The past tense was utilized commonly to present emotional-touching facts about the previous US presidents' failure in recognizing Jerusalem as the capital of Israel. The simple present was used to present his decision and his inclination as in ‘we want an agreement that is a great deal for the Israelis and a great deal for the Palestinians” (clause 44). The future time was used to communicate the emotion-touching impacts of his decision as in “But we are …cooperation” (clause 52).

In building *pathos*, President Trump began his clauses with adjuncts and subjects. He used the adjunct to precede the clauses for two main reasons: for cohesiveness (using conjunctive adjuncts ‘as’, ‘and’, ‘so’, etc.) and for emphasizing to the audience about the facts represented by the adjuncts (circumstantial, mood, comment). For example, in “While previous presidents …deliver” (clause 15). The comment adjunct (“While …promise,”) tells the audience that the previous presidents could only promise which implies they did not have a strong commitment to doing their best. This sentence was clearly used to arouse the negative sentiment of the audience toward the previous presidents/governments.

The element of complement also plays a vital role in constructing *pathos* in which the important words, either in the form of noun phrases or adjectives, convey important messages that may arouse the audience's emotional reaction. For example, in “Our children … our conflicts” (clause 56), the noun phrase ‘our love, not our conflicts’ gives an emotional touch. The use of contradictory words ‘love’ and conflicts' will easily arouse the audience's emotional reaction. President Trump seemed to assert to the audience indirectly that what he did was something related to ‘love’ not something that may inflict a ‘conflict’.

The same thing occurs in “Let us rethink …” (clause 66) where the complements (‘old assumptions’ and ‘our hearts and minds’) contain the power words to arouse the audience's emotional reaction. The phrase ‘old assumptions’ were used to degrade the policies undertaken by the previous governments as merely old assumptions and, therefore, should be forgotten. Another phrase, ‘our hearts and minds’, was clearly used to assert to the audience to use their hearts and minds in taking action. This phrase also implies that the previous presidents or governments did not use both of them to determine their policies which resulted in adverse impacts.

In appealing to the audience's logic (*logos*), President Trump used some clauses delivered in the declarative mood which functioned as a statement of fact. The mood type and speech-function realization of his *logos* clauses are described in Table 3.

**Table 3:** Mood type and speech-function realization in President Trump's *logos* clauses.

Mood type	Speech Function Realization	Technique	Example
Declarative	Statement of fact	Presenting a precedent	(j) *In 1995, Congress adopted the Jerusalem Embassy Act, urging the federal government to relocate the American embassy to Jerusalem and to recognize that that city — and so importantly — is Israel's capital. (clause 6)*
		Presenting details of the precedents	(k) *This act passed Congress by an overwhelming bipartisan majority. (clause 7)*
		Presenting the third-person opinion	(l) *Some say (that) they lacked courage, (clause 11)*
		Presenting a present fact	(m) *“Today, Jerusalem is the seat of the modern Israeli government.” (clause 25)*

In convincing the audience of the importance of recognizing Jerusalem as the capital city of Israel, he posed many logical reasoning and facts. The clauses were mainly delivered in the declarative mood to give the audience some information and claim some factual facts about the Jerusalem Embassy Act, the law's waiver, and Jerusalem. As can be seen in Table 3, four techniques were applied: Presenting a precedent, presenting details of the precedents, presenting the third person opinion, and presenting a present fact.

The first technique was presenting a precedent that was one of the important aspects of appealing to *logos* because it is commonly used as a justification of an argumentation. The precedents or some referential events in the past were posed by President Trump to support his deductive reasoning. For example, in (j), he posed a similar event in the past (Congress adopted the Jerusalem Embassy Act urging the federal government to recognize Jerusalem as the capital city of Israel) as the basis for his argumentation that Jerusalem should be recognized as the capital city of Israel. In other words, he would like to say that what he decided at that time was actually a manifestation of what had been decided by congress more than 20 years ago; therefore he had done the right thing on it.

The second technique is by presenting details of the precedent as in (k). In this instance, President Trump gave further information about the Jerusalem Embassy Act as a precedent mentioned earlier. He informed that the Act passed Congress by an overwhelming bipartisan majority. He used the clause – and the next one (clause 8) – to explain why the Act should be adopted by his government.

The third technique is presenting the third person opinion as in (l). Here, President Trump informed the audience of what the others say about the previous government. This clause was to support his argument that the previous presidents had failed to bring about peace. By presenting this clause, President Trump seemed objective about his judgment because the others agreed with him and had the same opinion.

The fourth technique is stating a present fact as in (m). In this instance, President Trump provided the audience with information about Jerusalem. He used the clause to support the decision he made. Because Jerusalem is the seat of the modern Israeli government, it is reasonable now to recognize Jerusalem as the capital city of Israel. The same clauses relating to this matter are clauses number 21, 26, 27, and 28. All the clauses talk about the present fact of Jerusalem.

In terms of the elements of mood, both personal and impersonal subjects were applied such as Israel, Jerusalem, US presidents, and congress. The word ‘Israel’ was used as the subject of the clause commonly to assert to the audience that Israel has the right to appoint Jerusalem as its capital city as in “…, Israel has made its capital in the city of Jerusalem” (clause 24). Interestingly, he used ‘previous US presidents’ as the subject of the clause to confirm the audience that they had made a fatal mistake (according to President Trump) that is by implementing the Jerusalem Embassy Act instead of adhering to law's waiver, as in “Yet, for over 20 years, every previous American president has exercised …” (clause 8).

In presenting *logos* to the audience, President Trump applied both positive and negative polarity in the finite. In talking about Israel and Jerusalem he commonly used positive polarity, while in talking about previous US presidents he often used negative polarity. The negative polarity was represented not only by ‘not’ but also by negative-sensed words as in (l). Interestingly, when talking about Israel and Jerusalem, he mostly used simple present which means the propositions are valid in the present time. In other words, he would like to say that anything about Israel and Jerusalem is the current fact as in “Today, …government” (clause 23).

President Trump commonly began his *logos* clauses with the elements of adjuncts and subjects. The high use of adjuncts in preceding the clauses is for two main reasons. Firstly, he would like to connect a clause with another clause for cohesiveness, using conjunctive adjuncts such as ‘but’, ‘nevertheless’, and ‘yet’. Secondly, he would like to emphasize to the audience about the facts represented by the adjuncts (circumstantial, mood, comment). For example, in clause 21, the clause-like adjunct “It was 70 years ago that” represented the fact of US's recognition of the State of Israel.

### 4.2 Discussion

In connection with the results of the previous researches outlined in the literature review, this study identifies three aspects of SFL in the speech that are interesting to discuss: the high use of declarative moods, various speech-function realizations, and negative elements in the clauses. Firstly**,** declarative moods strongly dominated the speech. Unlike Ayoola's research (2013) which showed many variations in the types of moods in political advertisements, the type of mood in President Trump's speech tended to be monotonous in which the declarative mood dominated the clauses. This finding indicates that in delivering his decision in the speech, the President positioned himself mostly as a carrier of information to his audience rather than as a requester of information (Eggins, 2004; Halliday, 2014).

The use of declarative mood helps him deliver his message (give information) directly without making a distance between him and the audience, as the nature of a declarative mood is to make a statement (Halliday, 2014). It is unlike the imperative or interrogative mood, for example, which tends to make a distance between the speaker and the hearer since they require the presence of the audience's responses to seeing whether the proposition is successful or not (Ayoola, 2013; Halliday, 2014). Hence, by applying the declarative mood, the message itself can be received instantly without requiring further thought and time from the side of the hearers. The President would like to assert that “It is the fact” or “it is true” which made the audience had no chance to challenge the information.

The information provided by President Trump through the declarative moods varied throughout the rhetorical elements of *ethos*, *pathos*, and *logos*. In the *ethos* clause, he gave information to the audience that his decision was true and unbiased. In his *pathos* clauses, he generally used the declarative moods to touch the audience's emotions, arousing both the audience's positive and negative feelings. In the *logos* clauses, he presented some facts underlying his decision.

Secondly, instead of functioning as statements, some declarative moods were used to make indirect requests to the audience which is one of the non-typical functions of a declarative mood (Eggins, 2004; Halliday, 2014), as exemplified in (h). This is in line with the result of Ayoola's research (2013) stating that the mood types in political discourse are not always in accordance with their typical speech functions. This finding is also following Wardhaugh's (2006) statement that a change of topic requires a change in the language used.

The mood types and, especially, the speech-function realizations also varied by the types of clauses (*ethos, pathos,* and *logos*). In President Trump's *ethos* clause, the mood type used was declarative which functioned as a statement of fact. In his *pathos* clauses, two types of mood were applied: declarative and imperative. The declarative moods had got different speech function realizations: statement of opinion, statement of assertion, statement of inclination, and indirect request. The imperative mood, however, functioned in line with its typical speech function, which is as a direct request. In his *logos* clauses, one type of mood was applied and functioned as a statement of fact.

Thirdly**,** one of the characteristics of President Trump's speech is the presence of many negative elements in the speech clauses, especially in the *pathos* and *logos*. His speech was like Ma's (Ko, 2015) in that many negative elements were present in the speech. The negative elements (blaming) were the element (the rhetorical device) that was not found in Obama's speeches, as revealed in the research conducted by Fengjie et al. (2016).

The negative element in President Trump's speech was in the form of blaming, especially of the former US presidents. He used many clauses containing negative evaluations (blaming) as the tool for persuading the audience. In this speech, blaming of the previous US presidents or governments were built either in the *pathos* or *logos* clauses, especially by raising fear and disgust in the audience toward the previous US presidents and government. According to Mulholland (1994), blaming (negative criticizing) is one of the tools for persuading the audience; because we tend to be polite and agree with the blamer (Hogan and Speakman, 2006). When something goes wrong, we often think that it is obviously the other person's mistake and that he/she should be blamed for the outcome.

The negative element was also reflected through the finite (polarity) of the clauses. President Trump used mostly positive polarity in building pathos or logos clauses, but in some clauses, he used the negative ones. When talking about the purpose of his decision, Israel, and the Middle East, he commonly used positive polarity; but when talking about previous US presidents, he frequently used negative polarity. He not only used ‘not’ to indicate negative polarity but also negative-sensed words as in “While previous presidents ... failed to deliver” (clause 15). The word ‘failed’ in this clause clearly reflects a negative sense (as opposed to ‘success’) because it refers to the unsuccessfulness of the previous presidents in solving the problem.

From the results of this current research, it can be concluded that the controversial side of President Trump's persuasive speech was not only because of its content which was actually controversial but was also because of choices of its mood types and speech function realizations. His many uses of clauses indicating a negative criticism (blaming) could ignite disagreement even unrest in the opponent side (the supporter of the former presidents). Being negatively criticized or blamed may cause fear, shame, or anger, and feeling worthless or incompetent (Greenberg, 2017). Therefore, the supporters of former presidents potentially got angry and shameful because they felt blamed. That is why the reaction from the opposite side also tended to be negative towards President Trump.

Additionally, Greenberg (2017) stated that blaming can be a way of asserting power and social control. This is well reflected in many clauses built for blaming others. In these clauses, President Trump presented to the audience that he as well as his government was very powerful and well-controlled the situation (e.g., clause 50, 70).

The results of this current research imply that public political speeches that contain many clauses of negative criticism toward political opponents have the potential to be a controversial speech. For the speaker's supporters, negative criticism towards the opposite side will make them more convinced that the policy being made was right and had to be met. For the opponent side, however, criticism will only make them increasingly dislike the speaker.

## 5 Conclusion

President Trump's speech of Jerusalem can be categorized as a persuasive speech whose primary purpose is to get the audience convinced and persuaded about the subject matter of the speech. His attempts to influence the audience can be seen from the grammatical choices he made. From the results of the study, it can be concluded that (1) the *ethos* clause was built by employing the declarative mood functioning as a statement to show his personal credibility; (2) the *pathos* clauses were composed by implementing two moods: mostly declarative, which mainly functioned as statements, and few imperative moods to arouse both positive and negative feeling of the audience; (3) and the *logos* clauses were composed by using the declarative moods functioning as statements to give bases for his argumentation. The high use of declarative moods indicated that he positioned himself as an information bearer, to shorten the gap between him and his audience.

In the grammatical perspective, the controversial side of the speech is mostly caused by the presence of many clauses containing negative elements (i.e., negative criticisms or blaming). Besides, the negative polarity of the clauses is evident in the speech, primarily when President Trump talked about previous US presidents and governments.

## Declarations

### Author contribution statement

A. Fanani: Conceived and designed the analysis; Analyzed and interpreted the data; Wrote the paper.

S. Setiawan: Conceived and designed the analysis.

O. Purwati: Analyzed and interpreted the data.

M. Maisarah: Contributed reagents, materials, analysis tools or data.

U. Qoyyimah: Analyzed and interpreted the data; Wrote the paper.

### Funding statement

This work was supported by LPDP (Indonesia Endowment Fund for Education), Ministry of Finance, Republic Indonesia [grant number 20161141080700].

### Competing interest statement

The authors declare no conflict of interest.

### Additional information

No additional information is available for this paper.

## Appendix The speech

Diplomatic Reception Room

1:07 P.M. EST

THE PRESIDENT: Thank you. When I came into office, I promised to look at the world's challenges with open eyes and very fresh thinking. We cannot solve our problems by making the same failed assumptions and repeating the same failed strategies of the past. Old challenges demand new approaches.

My announcement today marks the beginning of a new approach to conflict between Israel and the Palestinians.

In 1995, Congress adopted the Jerusalem Embassy Act, urging the federal government to relocate the American embassy to Jerusalem and to recognize that that city — and so importantly — is Israel's capital. This act passed Congress by an overwhelming bipartisan majority and was reaffirmed by a unanimous vote of the Senate only six months ago.

Yet, for over 20 years, every previous American president has exercised the law's waiver, refusing to move the U.S. embassy to Jerusalem or to recognize Jerusalem as Israel's capital city.

Presidents issued these waivers under the belief that delaying the recognition of Jerusalem would advance the cause of peace. Some say they lacked courage, but they made their best judgments based on facts as they understood them at the time. Nevertheless, the record is in. After more than two decades of waivers, we are no closer to a lasting peace agreement between Israel and the Palestinians. It would be folly to assume that repeating the exact same formula would now produce a different or better result.

Therefore, I have determined that it is time to officially recognize Jerusalem as the capital of Israel.

While previous presidents have made this a major campaign promise, they failed to deliver. Today, I am delivering.

I've judged this course of action to be in the best interests of the United States of America and the pursuit of peace between Israel and the Palestinians. This is a long-overdue step to advance the peace process and to work towards a lasting agreement.

Israel is a sovereign nation with the right like every other sovereign nation to determine its own capital. Acknowledging this as a fact is a necessary condition for achieving peace.

It was 70 years ago that the United States, under President Truman, recognized the State of Israel. Ever since then, Israel has made its capital in the city of Jerusalem — the capital the Jewish people established in ancient times. Today, Jerusalem is the seat of the modern Israeli government. It is the home of the Israeli parliament, the Knesset, as well as the Israeli Supreme Court. It is the location of the official residence of the Prime Minister and the President. It is the headquarters of many government ministries.

For decades, visiting American presidents, secretaries of state, and military leaders have met their Israeli counterparts in Jerusalem, as I did on my trip to Israel earlier this year.

Jerusalem is not just the heart of three great religions, but it is now also the heart of one of the most successful democracies in the world. Over the past seven decades, the Israeli people have built a country where Jews, Muslims, and Christians, and people of all faiths are free to live and worship according to their conscience and according to their beliefs.

Jerusalem is today, and must remain, a place where Jews pray at the Western Wall, where Christians walk the Stations of the Cross, and where Muslims worship at Al-Aqsa Mosque.

However, through all of these years, presidents representing the United States have declined to officially recognize Jerusalem as Israel's capital. In fact, we have declined to acknowledge any Israeli capital at all.

But today, we finally acknowledge the obvious: that Jerusalem is Israel's capital. This is nothing more, or less, than a recognition of reality. It is also the right thing to do. It's something that has to be done.

That is why, consistent with the Jerusalem Embassy Act, I am also directing the State Department to begin preparation to move the American embassy from Tel Aviv to Jerusalem. This will immediately begin the process of hiring architects, engineers, and planners, so that a new embassy, when completed, will be a magnificent tribute to peace.

In making these announcements, I also want to make one point very clear: This decision is not intended, in any way, to reflect a departure from our strong commitment to facilitate a lasting peace agreement. We want an agreement that is a great deal for the Israelis and a great deal for the Palestinians. We are not taking a position of any final status issues, including the specific boundaries of the Israeli sovereignty in Jerusalem, or the resolution of contested borders. Those questions are up to the parties involved.

The United States remains deeply committed to helping facilitate a peace agreement that is acceptable to both sides. I intend to do everything in my power to help forge such an agreement. Without question, Jerusalem is one of the most sensitive issues in those talks. The United States would support a two-state solution if agreed to by both sides.

In the meantime, I call on all parties to maintain the status quo at Jerusalem's holy sites, including the Temple Mount, also known as Haram al-Sharif.

Above all, our greatest hope is for peace, the universal yearning in every human soul. With today's action, I reaffirm my administration's longstanding commitment to a future of peace and security for the region.

There will, of course, be disagreement and dissent regarding this announcement. But we are confident that ultimately, as we work through these disagreements, we will arrive at a peace and a place far greater in understanding and cooperation.

This sacred city should call forth the best in humanity, lifting our sights to what it is possible; not pulling us back and down to the old fights that have become so totally predictable. Peace is never beyond the grasp of those willing to reach.

So today, we call for calm, for moderation, and for the voices of tolerance to prevail over the purveyors of hate. Our children should inherit our love, not our conflicts.

I repeat the message I delivered at the historic and extraordinary summit in Saudi Arabia earlier this year: The Middle East is a region rich with culture, spirit, and history. Its people are brilliant, proud, and diverse, vibrant and strong. But the incredible future awaiting this region is held at bay by bloodshed, ignorance, and terror.

Vice President Pence will travel to the region in the coming days to reaffirm our commitment to work with partners throughout the Middle East to defeat radicalism that threatens the hopes and dreams of future generations.

It is time for the many who desire peace to expel the extremists from their midst. It is time for all civilized nations, and people, to respond to disagreement with reasoned debate –- not violence.

And it is time for young and moderate voices all across the Middle East to claim for themselves a bright and beautiful future.

So today, let us rededicate ourselves to a path of mutual understanding and respect. Let us rethink old assumptions and open our hearts and minds to possible and possibilities. And finally, I ask the leaders of the region — political and religious; Israeli and Palestinian; Jewish and Christian and Muslim — to join us in the noble quest for lasting peace.

Thank you. God bless you. God bless Israel. God bless the Palestinians. And God bless the United States. Thank you very much. Thank you.

(The proclamation is signed.)

END

1:19 P.M. EST
